# Activation of Nrf2-Antioxidant Response Element Mediated Glutamate Cysteine Ligase Expression in Hepatoma Cell line by Homocysteine

**DOI:** 10.5812/hepatmon.8394

**Published:** 2013-04-20

**Authors:** Monireh Mani, Shahnaz Khaghani, Taghi Gol Mohammadi, Zahra Zamani, Kayhan Azadmanesh, Reza Meshkani, Parvin Pasalar, Ehsan Mostafavi

**Affiliations:** 1 Department of Biochemistry, School of Medicine, Tehran University of Medical Science, Tehran, IR Iran; 2 Department of Biochemistry, Pasteur Institute, Tehran, IR Iran; 3 Department of Virology, Pasteur Institute, Tehran, IR Iran; 4 Department of Epidemiology, Pasteur Institute, Tehran, IR Iran

**Keywords:** HepG2 Cells, Oxidative Stress, Homocysteine

## Abstract

**Background:**

Homocysteine is a sulfur-containing amino acid which formed (mainly in the liver) during the metabolism of methionine. Prior studies indicated the important role of hyperhomocysteinemia in pathogenesis and progression of alcoholic liver disease, liver steatosis and cirrhosis. One of the most important mechanisms by which homocysteine promote the development of hepatic injury is oxidative stress. Transcription factor Nrf2-mediated antioxidant response, represents critical cellular defense mechanism that serves to maintain intracellular redox homeostasis and limit oxidative stress. Glutamate cysteine ligase catalytic (GCLc) is rate limiting enzyme in the synthesis of glutathione, an important endogenous antioxidant.

**Objectives:**

This study was conducted to investigate whether homocysteine induces the Nrf2 dependent expression of GCLc in hepatoma cell line (HepG2) and whether this induction is mediated by antioxidant response element (ARE) which present within its promoter.

**Materials and Methods:**

Glutathione (GSH) content was measured by flow cytometry. Using electro mobility shift assay (EMSA) and western blotting, ARE-binding activity of Nrf2 for GCLc was demonstrated. Real time RT-PCR and western blotting were performed to evaluate whether homocysteine was able to induce mRNA and protein expression of GCL.

**Results:**

Exposure of HepG2 cells to 50 µMD/L homocysteine and western blotting of nuclear extracts revealed that Nrf2 is strongly stabilized and became detectable in nuclear extracts. EMSA demonstrated increased binding of Nrf2 to oligomers containing GCL promoter - specific ARE -binding site.A time- dependent increase in the gene and protein expression of GCL was observed. Additionally, GSH, which is prime scavenger of free radicals in cells, decreased initially. Elevation of GSH, following the initial decline, closely correlated with gene expression profile of GCLc, which is a rate-limiting enzyme in GSH synthesis.

**Conclusions:**

Altogether, we provide direct evidence that homocysteine activates Nrf2-mediated antioxidant response, which protects HepG2 cells from oxidative damage.

## 1. Background

Hyperhomocysteinemia (HHcy) has been implicated as a risk factor for atherosclerosis, cerebrovascular and peripheral vascular diseases (1-3). An increasing body of data links HHcy to hepatic dysfunctions. Elevation of homocysteine levels due to insult in homocysteine metabolism is observed in patients with liver cirrhosis and chronic alcohol consumption (4, 5). The enzymes 5,10-methylenetetrahydrofolate reductase (MTHFR) and cystathionine β synthase (CBS) play critical roles in regulating plasma homocysteine. Nutritional deficiency and mutations in enzymes which remove homocysteine lead to HHcy (6, 7). One of the clinical manifestations of the patients with CBS and MTHFR deficiency is hepatic steatosis and fibrosis (8-10). The underlying mechanisms contributing the pathogenesis of liver injury to HHcy are still poorly understood and may be multifactorial. Studies on the mechanisms of homocysteine associated disorders have suggested that homocysteine induced Oxidative stress might play a major role in the pathology of damage (11-13). Induction of glutamate cysteine ligase catalytic (GCLc) which is a member of a family of antioxidant/detoxification enzymes is important to maintain cellular redox homeostasis and reduce oxidative damage. GCLc is a rate-limiting enzyme in glutathione (GSH) synthesis. Induction of this enzyme is through a cis-acting element in the promoter region known as the antioxidant response element (ARE). AREs are known to play essential roles in regulating the cellular responses to oxidative stress (14, 15). GSH is a major intracellular antioxidant in mammals, and the liver is the major site of its synthesis from the point of view of quantity (16). In cells exposed to oxidative stress, expression of the nuclear factor erythroid-derived 2-like 2 (Nrf2) is enhanced. Nrf2 positively regulates the transcriptional activation of a number of genes in that their expression is essential to protect cells against loss of viability under homeostatic conditions (17). There has been no assessment as to whether Nrf2 is involved in the oxidative stress response induced by homocysteine in liver and a direct link between homocysteine mediated GCLc induction and activation of Nrf2 expression via stimulation of ARE binding activity remains to be examined.

## 2. Objectives

In this study, we analyzed the involvement of Nrf2 and ARE activation in GCL induction by homocysteine in hepatoma cell line.

## 3. Materials and Methods

### 3.1. Study design

This experimental study was performed to determine the effect of homocysteine on mRNA and protein expression of HO-1 in HepG2 cells and the role of Nrf2-ARE pathway in this induction. HepG2 cells were cultured by routine method. The cells were divided into two groups: control and treated with homocysteine. HO-1 expression was measured by real time PCR and protein expression by western blotting. Glutathione content was examined by flowcytometry.

### 3.2. Cell Culture, Treatment and Cell Viability Assay

The HepG2 cells (National Cell Bank, Pasteur Institute of Iran) were cultured in Dulbecco's modified Eagle's medium (DMEM) supplemented with 10 % fetal calf serum and 100 u/ml penicillin G sodium, 100 µg/ml streptomycinand L-glutamine in humidified atmosphere in 5% CO2 at 37ºC. For RNA extraction and glutathione detection, the cells were seeded at the density of 5 × 10^5^ per well in 6 well culture plates. For isolation of whole cell and nuclear protein extract, cells were plated at a density of 2 × 10^6^ in 25 cm^2^ flasks. The culture media were replaced with fresh media before different treatments. The untreated cells were used as controls. The cells were treated for 15 min, 30 min, 45 min, 3h, 6h and 9h with homocysteine. Homocysteine (Sigma Chemical Co, USA) was used as D/L homocysteine at a final concentration of 50 µM, which correspond to 25 µML homocysteine. For cell viability assay HepG2 cells (1 × 10^6^ cells/ml) were incubated in control or test medium supplemented with 5% fetal calf serum at 37°C for 18 h. Cell viability was assessed by counting cells on a hemocytometer after suspension in PBS (pH = 7) containing 0.5% trypan blue stain. Data shows the means ± SD from three independent experiments.

### 3.3. Intercellular Glutathione Content

Glutathione content of the cells was measured using the ThiolTracker Violet GSH detection reagent (Invitrogen/Molecular probes, Eugene, OR) according to the manufacturer's protocol. Cells were seeded in six-well plates, incubated overnight and treated the following day. Subsequent to homocysteine exposure for the indicated times (15 min,30 min,45 min, 3h,6h,9h), the cells were washed with PBS and incubated in Dulbecco's PBS containing 10 µM (final concentration) ThiolTracker Violet for 30 min at 37ºC. Then cells were analyzed on a Flow cytometer (Partec, Germany). Analysis of cellular GSH content was restricted to PI negative intact cells.

### 3.4. Preparation of Total RNA and Real Time Quantitative RT-PCR

Total RNA was isolated from HepG2 cells using Rneasy cell mini kit (QIAGEN GmbH, Hilden, Germany) according to the manufacturer's protcol. Quantification and purity of RNA samples were assessed by A260/A280 absorption, and RNA samples with ratios above 1.8 were stored at -70°C for further analysis. Three micrograms of total RNA were subjected to reverse transcription using Quantitect Revese Transcription Kit (Fermentase, Vilnius, Lithuania). Real-time quantitative RT-PCR was done using SYBR premix Taq (Takara, Japan) and primer for GCLc (QT00037310, QIAGEN GmbH, Hilden, Germany). The transcript for the constitutive gene product β-actin (QT01680476, QIAGEN GmbH, and Hilden, Germany) was used as endogenous control for data normalization. Each sample type was run in duplicate and each experiment was performed four times. Each cycle consisted of denaturation at 95ºC for 5sec and combined annealing and extension at 60ºC for 30sec. The expression levels of the gene were quantified with REST 2009 software.

### 3.5. Preparation of Protein Extract for Western Blotting

For preparation of nuclear extracts, cells were homogenized in lysis buffer, consisting of 10 mM HEPES (pH = 7.9),1.5 mM MgCl_2_,10 mM KCl,0.5 mM dithiothreitol (DTT) and protease inhibitor cocktail (Roche, Mannheim, Germany). Subsequently, homogenate was then centrifuged at 3000 rpm for 10 min at 4ºC. The resulting nuclear pellet was resuspended in 30 µl of cold buffer B, consisting of 5 mM HEPES (pH = 7.9),1.5 mM MgCl_2_,0.2 mM EDTA,0.5 mM DTT, 26% glycerol and protease inhibitor. The suspension underwent centrifugation at 14800 g for 5 min at 4ºC. The protein concentration was measured by the Bradford method. For preparation of whole cell extract, cells were lysed in RIPA lysis buffer (150 mM sodium chloride, 1% Np-40, 0.5% sodium deoxycholate,0.1% SDS,50 mM tris pH = 8) and protease inhibitor cocktail on ice. Lysates were incubated on ice for 10 min at 4ºC and subsequently centrifuged at 9300 g for 10 min. Finally, supernatants were saved and stored at -70ºC. The amount of protein was measured by the Bradford method.

### 3.6. Western Blotting Analysis

Sample proteins from whole cell lysates (30 µg) and nuclear extracts (40 µg) were loaded on sodium dodecyl sulfate polyacrylamide gels (8% and 10% respectively). Separated proteins by SDS-PAGE were transferred to a nitrocellulose membrane and blocked. Immunoblot analysis was carried out using the following primary antibodies; anti GCLc (1/400) polyclonal antibody (abcam Co, UK), antiNrf2 (1/500) polyclonal antibody (abcam Co, UK). Antiβ-actin (1/3000) polyclonal antibody and antilamin B (1/2000) polyclonal antibody (abcam Co, UK) were used for normalization of the data. HRP-conjugated goat-anti rabbit IgG were used as secondary antibody. Blots were developed using the enhanced chemiluminescence immunoblot detecting reagent (ECL plus Western blotting detection system, GE Healthcare). The intensity of bands was calculated with image J software (version 1.46a).

### 3.7. Electrophoretic Mobility Shift Assay (EMSA)

Nuclear extracts were prepared as described above. The synthetic complementary oligonucleotides 5'-GCACAAAGCGCTGAGTCACGGGGAGG-3'and 5'-CCTCCCCGTGACTCAGCGCTTTGTGC-3' (sigma, USA) were 3'-biotinylated using the biotin 3'-end DNA labeling Kit (pierce) according to the manufacturer's instructions. The standard binding reaction mixture contained 50 ng/µl poly (dI-dc),0.05% Nonidet P-40,5 mM MgCl_2_,10 mM EDTA, and 2.5% glycerol in 1X binding buffer (Lightshift chemiluminescent EMSA kit, Pierce), 20 f M of biotin-end -labeled target DNA and 10 µg of nuclear extract. The reaction mixture was incubated at 25ºC for 20 min. The DNA-protein complex was separated in a 5% polyacrylamide gel in 0.5X Tris borate/EDTA, and then transferred on to a positively charged nylon membrane in 0.5 X Tris borate/EDTA at 100V for 30 min. The membrane was UV-cross-linked and the biotin end-labeled probe was detected with streptavidin-HRP using a luminol enhancer solution (Lightshift chemiluminescent EMSA kit) according to the manufacturer's instruction. For supershift assays, 1 µl of 1 µg/µl Nrf2 antibody (SANTA CRUZ SC- 722 X) was added to the reaction mixture and incubated for 30 min at room temperature prior to electrophoresis. A 200-fold excess of unlabeled oligonucleotide (competitor) was added where necessary.

### 3.8. Statistical Analysis

SPSS version 16 was used for data analysis. Data were summarized using descriptive statistics (means ± standard error). Significance of differences between control and treated groups was determined by one-way analysis of variance (ANOVA). LSD post-hoc analysis was performed for comparing the groups. For all analyses, P value less than 0.05 were considered significant.

## 4. Results

### 4.1. Effect of Homocysteine on the Viability of HepG2 Cells

To examine the effect of homocysteine on the viability of human hepatoma cells, HepG2 cells were incubated with 50 µMD/L homocysteine for up to 18 h. Exposure of HepG2 cells (1 × 10^6^ cells/ml) to 50 µM homocysteine had no effect on cell viability [Control (live cells: 93 ± 0.7) vs. treatment group with 50 µM homocysteine (live cells: 93 ± 1.0)] (P = 0.85).

### 4.2. Homocysteine Alters GSH Level

**Figure 1.**
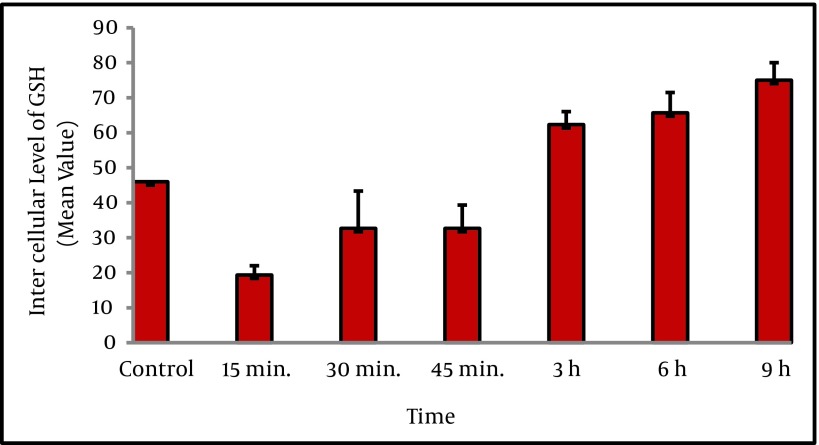
Intercellular Levels of GSH Using Flow Cytometry in HepG2 Cells Exposed To Homocysteine HepG2 cells were treated with D/L homocysteine (50 µM) for the indicated times, and harvested for flow cytometric analysis of intracellular glutathione content using mean channel fluorescence (MCF) of ThiolTracker Violet dye (TTV). Analysis of cellular GSH content was restricted to PI negative intact cells. Values are means ± SE. Statistically different from control.

The intracellular GSH level was determined following treatment with homocysteine. Changes in intracellular GSH exhibited two phases (Figure 1). First, GSH content from 46% in untreated cells significantly decreased to about 19% and 35% within 15 min and 45 min of homocysteine exposure respectively. Then, the depleted GSH level began to recover and reached to 60% at 3h, and 76 % (maximum level) at 9h. This reversal is likely caused by increased GCLc levels.The statistical analysis showed that there was a significant difference between GCLc protein level at 6 h and 9h with controls (P < 0.001). The statistical analysis also showed that there was a significant difference between GCLc protein level in different treatment time periods (P < 0.001).

### 4.3. Homocysteine Induces GCLc mRNA and Protein Expression

**Figure 2.**
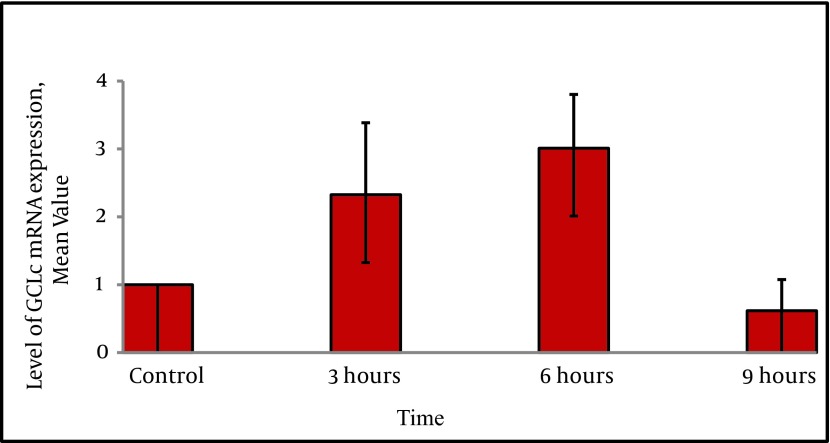
Homocysteine Induces GCLc mRNA Expression HepG2 cells were treated with D/L homocysteine (50 µM) for the indicated time points and GCLc mRNA levels were determined using quantitative real-time RT-PCR analysis. The bar graph shows the quantization of GCLc gene expression. Values are normalized to β-actin expression and represent the means ± SE of four separate experiments.

To evaluate whether homocysteine was able to induce GCLc mRNA expression, cells were treated with homocysteine and quantitative real-time RT-PCR analysis was performed.The inducing effect of homocysteine was observed after 3h and 6h of treatment. Levels of GCLc mRNA expression were increased 2.3 fold ± 0.91 and 3 fold ± 0.68 after 3h and 6 h respectively (Figure 2). The statistical analysis showed that there was a significant difference between expression levels at 3h and 6h with control (P < 0.05). The statistical analysis also showed that there was a significant difference between expression levels in different treatment time periods (P = 0.004).

**Figure 3.**
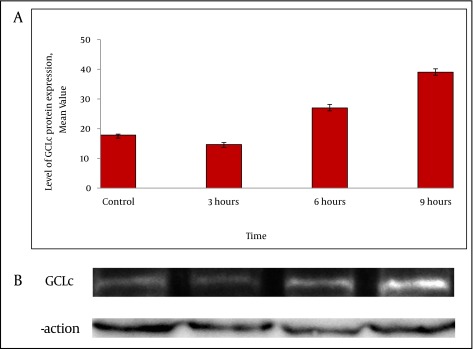
GCLc Protein Expression in HepG2 Cells Exposed to Homocysteine A) The protein levels were detected by western blotting. Values represent the means ± SE of three separate experiments. The bar graph shows the quantization of GCLc protein B) Western blot analysis of GCLc in HepG2 cells. The cells were incubated with 50 μM homocysteine for 3, 6 and 9 h, and the cell extracts were electrophoresed, protein transferred, and blotted with the polyclonal antibody to GCLc. GCLc is a major band appearing at approximately 73KD. β-actin was used as loading control.

To confirm the results obtained from real time RT-PCR experiment, western blot analysis was performed. The levels of GCLc protein expression started to decline 3h after exposure to homocysteine and then increased by 1.6 fold and 2.3 fold in HepG2 cells treated with homocysteine for 6h and 9 h respectively (Figure 3).

### 4.4. Homocysteine Increases the Nrf

**Figure 4.**
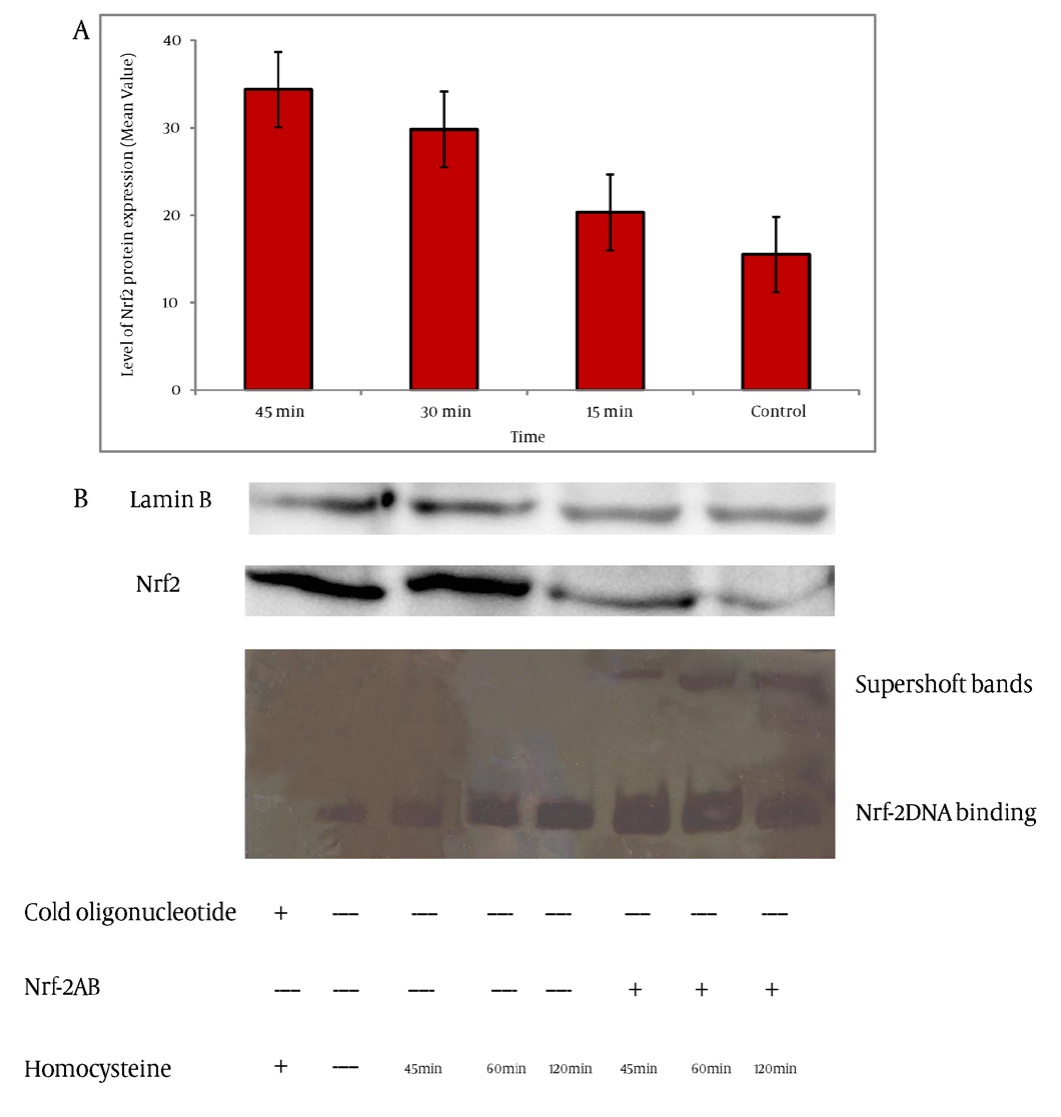
Homocysteine Activates Nrf2-Mediated Antioxidant Response A) Nrf2 protein levels in nuclear fractions of HepG2 cells exposed to homocysteine (50 µM) for the indicated time. The nuclear cell extracts were electrophoresed, protein transferred, and blotted with the polyclonal antibody Nrf2. Nrf2 is a major band appearing at approximately 68 kD. Lamin B was used as loading control. Quantification of band intensity was performed by Image J (version 1.46a). The bar graph shows the quantization of Nrf2 protein. Values represent the means ± SE of three separate experiments B) Gel shift assay using oligomers containing the GCLc promoter-specific ARE-biding site and nuclear fractions of HepG2 cells that were exposed to homocysteine for the indicated time periods. Competitor (200 fold excess) and Nrf2 antibody were applied as indicated.

Incubation of the nuclear extracts after treatmentwith homocysteine, with ARE oligomers containing the GCLc promoter specific Nrf2-binding site resulted in the enhanced GCLc ARE-binding activity of Nrf2. The ARE-binding complex was increased at 45 min of homocysteine treatment and was maintained for 120 min (Figure 4b). Densitometric evaluation showed that levels of nuclear DNA binding of Nrf2 was increased by 1.4 fold, 2.4 fold and 3.8 fold after 45 min, 60 min and 120 min of treatment with homocysteine respectively. Addition of an anti-Nrf2 antibody indicates involvement of the Nrf2 transcription factor at this site. There was a significant difference between binding nuclear protein level at 1h and 2h compared to that of the control (P < 0.001).

### 4.5. Homocysteine Stimulates the Nrf

The nuclear translocation of Nrf2 is an important mechanism that contributes towards gene expression. To explore whether homocysteine stimulated nuclear translocation of Nrf2, western blot analysis of nuclear extract was conducted. As shown in Figure 4a, homocysteine increased the nuclear accumulation of Nrf2, which reached a maximum at 45 min after homocysteine treatments. Densitometric evaluation showed that levels of nuclear Nrf2 increased by 1.3 fold, 1.92 fold and 2.21 fold in HepG2 cells treated with homocysteine after 15 min, 30 min and 45 min in compared to that of the control.

## 5. Discussion

Hyperhomocysteinemia has been suggested to have a role in pathogenesis of various diseases including hepatic dysfunction. Patients with hyperhomocysteinemia can develop hepatic steatosis and fibrosis (8-10). Previous studies have suggested that the pathophysiological effects of homocysteine are mediated via oxidative stress (18, 19) and oxidative stress plays an important role in different types of liver injury (20, 21). In the present study, we used HepG2 cells as a cellular model to consider the role of the self-protecting systems in the cells arise upon oxidative stress induced by homocysteine. We used 50 µMD/L homocysteine for treatment of the cells. This concentration of homocysteine can be found in hyperhomocysteinemic individuals (22). This concentration did not affect overall cell number or viability as determined by trypan blue assay. In the current study, we found that alterations of intracellular GSH caused by homocysteine exposure exhibited two phases, an elevation of intracellular GSH, following the initial decline within the first hour of homocysteine exposure. This initial decline might indicate that, to combat against oxidative stress induced by homocysteine, GSH might be the first line of antioxidant defense. We also found that, following the depletion of GSH, a compensatory increasing in GCLc mRNA and protein occurs. Using quantitative real time RT PCR analysis and western blotting, an increase in mRNA and protein levels for catalytic subunit of GCL could be demonstrated. This may at least in part be due to the relief in the feedback inhibition of the catalytic subunit of GCL and induction of this enzyme (23). The elevation of intracellular GSH following its initial decline, well correlated with increased expression of the GCLc which is a key antioxidant enzyme in regulation of GSH synthesis. Since the enzymes regulating GSH synthesis such as GCLc, are regulated at least in part by Nrf2, these results provided the direct evidence that Nrf2 can be activated by homocysteine. To elucidate the role of Nrf2 in transcriptional activation of ARE in response to homocysteine, electro mobility shift assay was performed. Using EMSA, we were able to demonstrate that homocysteine induces increased binding of nuclear proteins in HepG2 cells to ARE- containing oligonucleotide. This is in agreement with a recent study where homocysteine induced increased binding activity of the GCL-ARE in mouse macrophages (24). Consistent with the results of EMSA, western blot analysis showed that, Nrf2 nuclear proteins accumulation was increased after stimulation with homocysteine. This revealed that, transcriptional factor Nrf2 plays a critical role in cellular response against homocysteine induced stress oxidative. Our data also indicates that Nrf2 is rapidly stabilized and mobilized to the nucleus in response to homocysteine treatment. Induction of GCL expression under regulation of Nrf2-ARE is consistent with observations of Huang et al, who reported that upregulation of GCL expression inhibited oxidative stress in association with increased Nrf2-ARE binding activity in hepatocytes (25). These results allow us to assemble a series of consequential events as follows: exposure to homocysteine causes GSH depletion that might change the redox status of the cell. This condition might trigger nuclear translocation of Nrf2 and activation of the Nrf2/ARE pathway to up-regulation of enzymes responsible for the synthesis of endogenous antioxidants such as GSH. This auto regulation mechanism could be proposed as a therapeutic target to prevent or reduce stress oxidative induced by homocysteine using pharmaceutical or natural dietary agents. In this context, several dietary compounds have been reported to increase Nrf2 levels, disassociate Nrf2 of its cytosolic inhibitor Keap1 or enhance Nrf2 protein stabilization, resulting in the activation of Nrf2-dependent gene expression (26, 27). In conclusion, our study showed that homocysteine induces the expression of GCL in HepG2 cells and this induction involves ARE within GCL promoter. We have also shown that this induction is dependent on Nrf2 nuclear accumulation. Increased expression of antioxidant genes via ARE-driven gene regulation could help to protect liver against homocysteine induced oxidative stress. As Nrf2/ARE pathwayshave been shown to provide protection against several oxidative impact in different organs (28-30) and increased expression of GCL decreases fibrogenic response in liver (31). Therefore, targeting of this pathway may be considered as a therapeutic strategy for the protection against oxidative liver damage caused by homocysteine, especially in cases with severe hyperhomocysteinemia due to enzymatic deficiencies accompanied with progressive hepatic steatosis and fibrosis.
